# Pitfalls of diffusion-weighted imaging of the female pelvis

**DOI:** 10.1590/0100-3984.2016.0208

**Published:** 2018

**Authors:** Ana Luisa Duarte, João Lopes Dias, Teresa Margarida Cunha

**Affiliations:** 1 Department of Radiology, Hospital do Espírito Santo E.P.E., Évora, Portugal.; 2 Department of Radiology, Hospital de São José, Centro Hospitalar de Lisboa Central, Lisboa, Portugal.; 3 Department of Radiology, Instituto Português de Oncologia de Lisboa Francisco Gentil, Lisboa, Portugal.

**Keywords:** Diffusion magnetic resonance imaging, Magnetic resonance imaging, Pelvis/diagnostic imaging

## Abstract

Diffusion-weighted imaging (DWI) is widely used in protocols for magnetic resonance imaging (MRI) of the female pelvis. It provides functional and structural information about biological tissues, without the use of ionizing radiation or intravenous administration of contrast medium. High signal intensity on DWI with simultaneous low signal intensity on apparent diffusion coefficient maps is usually associated with malignancy. However, that pattern can also be seen in many benign lesions, a fact that should be recognized by radiologists. Correlating DWI findings with those of conventional (T1- and T2-weighted) MRI sequences and those of contrast-enhanced MRI sequences is mandatory in order to avoid potential pitfalls. The aim of this review article is the description of the most relevant physiological and benign pathological conditions of the female pelvis that can show restricted diffusion on DWI.

## INTRODUCTION

The use of diffusion-weighted imaging (DWI) in magnetic resonance imaging (MRI) of the pelvis has become more widespread in recent years. Stronger diffusion gradients and faster imaging techniques have been developed, particularly after the introduction of parallel imaging, which provided high-quality DWI of the body with substantially fewer motion artifacts^(^^1-9^^)^.

DWI of the pelvis is now routinely used, allowing tissue characterization at a microscopic level, based on the Brownian motion of water molecules, when two diffusion gradients are added to T2-weighted (T2W) sequences^(^^1,2,5-7,9-13^^)^. Those two gradients allow the characterization of tissues by respectively dephasing and rephasing the water molecules, the movement of which is restricted by the presence of cell membranes and macromolecules. If a tissue has low cellularity and defective cell membranes, the water molecules will move more "freely"; when the first gradient is applied they will acquire a different phase but will maintain their "free" movement when the second gradient is applied, leading to signal loss because they are not perfectly rephased by the second gradient. If a tissue is highly cellular, with intact cell membranes, the movement of the water molecules will be "restricted" and they will not have moved substantially between the first and second gradient, hence the second gradient will "cancel out" the first one and the T2 signal of the tissue will be maintained. Therefore, the reduction of signal intensity (SI) in DWI represents the movement of water molecules. The more "freely" water molecules move, the greater will be the signal loss^(^^1,2,7,12,14^^)^. The diffusion gradients applied have different b values, which translate to different strengths. At low b values (e.g., b = 50-100 s/mm^2^), there will be signal loss for highly mobile water molecules, such as those within the vessels, producing the so-called "black-blood" effect. At higher b values (e.g., b = 500-1000 s/mm^2^), the true diffusion of a tissue is shown, because the more "restricted" the water molecule movement is, the stronger will be the signal emitted by those molecules^(^^1-3,10,15^^)^. Therefore, tissues with high cellularity, such as tumor tissue, will consistently show high SI on DWI, especially at high b values^(^^2,3,5,16^^)^.

For an accurate DWI analysis, parametric maps of the apparent diffusion coefficient (ADC), based on at least two different b values, are created for each voxel of an image. These ADC maps are independent of magnetic field strength and show the different tissue diffusion properties at different b values, displaying them in gray-scale images^(^^1,2,7,12-14^^)^. Areas with "free" water molecule movement will show high SI on ADC maps and low SI on DWI with high b values, whereas areas with restricted diffusion, such as tumor tissue, will show low ADC values ("darker" images) and high SI at high b values^(^^3,5-7,10,11,14,16^^)^. The ADC maps are also useful to avoid a common DWI pitfall, the so-called "T2 shine-through" effect. Because DWI is based on T2W images, tissues with a very long T2 relaxation time, such as simple cysts, can show high SI at all b values, including 1000 s/mm^2^. However, the high SI on DWI of such tissues does not indicate "true" restricted diffusion, because the ADC map will also demonstrate high values^(^^1-7,10,12-14,16^^)^.

**Figure 1:**
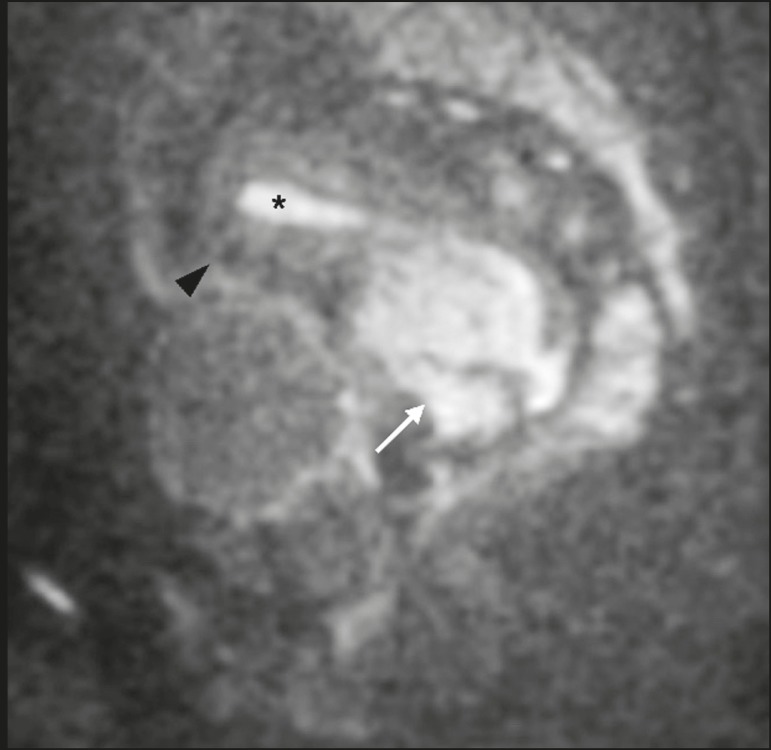
MRI of a female patient with cervical cancer. Sagittal DWI at b = 1000 s/mm^2^ showing the low SI of the myometrium (arrowhead), the high SI of the endometrium (asterisk), and the high SI of the tumor (arrow).

High SI on DWI accompanied by low SI on ADC maps is usually associated with malignant tumors (Figure 1). However, when interpreting DWI scans of the female pelvis, radiologists should be aware that 22% of lesions that exhibit restricted diffusion are benign, whether they are cystic, such as abscesses, or solid, such as cellular leiomyomas^(^^5,13,15^^)^. The aim of this review was to present the benign physiological and pathological conditions of the female pelvis that can show restricted diffusion on DWI.

## UTERUS

Because of its high proportion of collagenous tissue, the normal myometrium-especially the junctional zone-shows low SI at any b value on DWI and discrete low SI on the ADC map. In contrast, the endometrium has relative high protein content and cellularity, typically showing high SI, at any b value, on DWI (Figure 1) and relative low SI on the ADC map^(^^4,12,14,16^^)^. The myometrium and endometrium differ in terms of their ADC values throughout the menstrual cycle and menopause. In the endometrium, ADC values are lower in the menstrual phase and in menopausal women, whereas they are higher in the proliferative phase and even higher in the secretory phase. In the myometrium, ADC values are lower in menopausal women and in the proliferative phase, whereas they are higher in the secretory phase and even higher in the menstrual phase. The ADC values are also different for women using oral contraceptives, the myometrium showing higher ADC values in women who take oral contraceptives than in women of reproductive age who do not^(^^17,18^^)^. DWI is now widely used for the assessment and staging of endometrial and cervical neoplasms, which tend to show typical restriction patterns^(^^3,4,6,19^^)^. However, radiologists should be aware of some potential pitfalls of using DWI to evaluate the uterus, exercising caution in order to avoid mistaking benign conditions for malignancy.

## CELLULAR LEIOMYOMAS VERSUS UTERINE SARCOMAS

**Figure 2:**
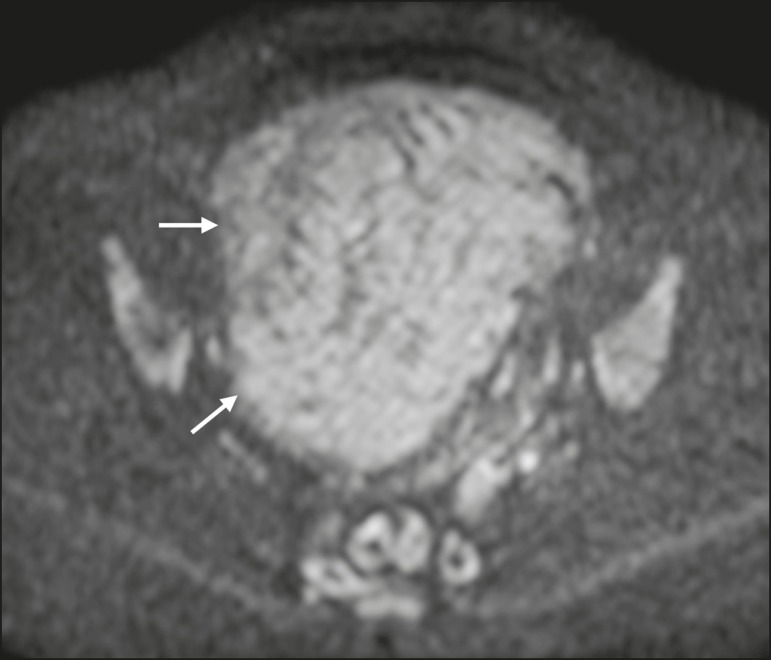
MRI of a female patient with cellular leiomyoma. Axial DWI at b = 1000 s/mm^2^ showing a large uterine tumor with high SI (arrows).

**Figure 3:**
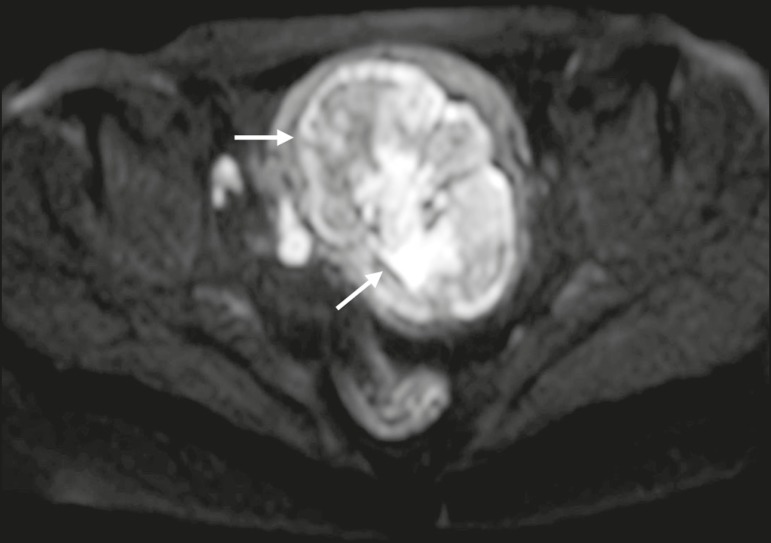
MRI of a female patient with leiomyosarcoma. Axial DWI at b = 1000 s/mm^2^ showing a large uterine tumor with high SI (arrows).

Leiomyomas, the most common myometrial tumors, are benign tumors that are usually easily diagnosed on MRI. They appear as well-circumscribed nodules that are hypointense on T2W and T1-weighted (T1W) images^(^^3,13,19,20^^)^. Because DWI is based on T2, leiomyomas tend to show low SI at various b values, even 1000 s/mm^2^, and accordingly low SI on the parametric ADC map: the so-called "T2 blackout" effect. This can be confusing because, as we mentioned above, dark ADC lesions can be considered suspicious. In fact, it emphasizes the importance of always analyzing DWI scans and the ADC map^(^^3,4,6,8,21-23^^)^. The cellular leiomyoma type, which is composed of densely cellular fascicles of smooth muscle with little intervening collagen, deserves particular attention. Cellular leiomyoma shows few mitotic figures and little or no cytological atypia. Due to its high cellularity, this type of leiomyoma does not show the classic MRI features on morphological sequences and can show an increased signal on T2W images^(^^4,6,19,20,23^^)^. On DWI, cellular leiomyomas can also display different features, including high SI at high b values (Figure 2) and low SI on the ADC map-behaving like malignant tumors do^(^^12,20,23^^)^. In rare cases, a leiomyoma can undergo sarcomatous transformation into a leiomyosarcoma, the most common malignant tumor of the myometrium^(^^4,6,19,23^^)^. Leiomyosarcomas appear as large heterogeneous tumors, infiltrating the adjacent myometrium, with intermediate to high SI on T2W images and low to intermediate SI on T1W images^(^^3,4,6^^)^. On DWI sequences, leiomyosarcomas have high SI at b = 1000 s/mm^2^ (Figure 3) and low SI on the ADC map-just like cellular leiomyomas^(^^7,13,23^^)^. Therefore, there is significant imaging overlap between cellular leiomyomas and leiomyosarcomas, which means that neither morphological sequences nor DWI are able to exclude malignancy^(^^3,4,6,7,13,23^^)^.

## ADNEXA

Radiologists should be aware of the fact that normal ovaries can have a relative high SI on DWI sequences at any b value, especially during the luteal phase. In addition, follicle cysts (which can reach up to 5 cm in diameter) can present with high SI in all DWI sequences, even those with high b values, and concomitant high SI on the ADC map-i.e., the "T2-shine through" effect^(^^24^^)^. When it comes to adnexal lesions, transvaginal ultrasound continues to be the first-line imaging modality, because it is affordable, is fast, and efficiently characterizes most such lesions^(^^4-6,8,9,19,25-31^^)^. However, MRI plays a vital role in the characterization and surgical planning of lesions classified as indeterminate on ultrasound^(^^3,8,9,19,27-29,32,33^^)^. DWI has been widely included in MRI protocols and has increased the accuracy for malignancy detection. Whereas ovarian malignancies tend to show solid areas with intermediate SI on T2W images and restricted diffusion, benign ovarian tumors are more likely to exhibit low SI on T2W images and low SI at high b values. Thomassin-Naggara et al.^(^^28^^)^ demonstrated that low SI on a T2W image of a solid portion of an ovarian lesion is a strong indicator of benignity, as has been shown by other authors^(^^4,8,13,16,26,29^^)^.

There is an overlap between benign and malignant ovarian tumors-restricted diffusion is not exclusive of malignant lesions. Features characteristic of malignancy on DWI can be seen in patients with ovarian torsion, abscess, endometrioma, hemorrhagic cyst, mature cystic teratoma, and non-edematous fibroma, thus creating potential pitfalls^(^^3,6,13,20,21,25,29,33-37^^)^.

## OVARIAN TORSION

Ovarian torsion is a serious cause of lower abdominal pain that can occur at any age, although it is more common in women of reproductive age. It can occur in women with an ipsilateral tumor or cyst (in 50-81% of cases), as well as in normal ovaries with long mesovaria^(^^32,34,35^^)^. The torsion initially causes venous stasis, which can progress to arterial stasis because of the edema. Complete arterial torsion results in hemorrhagic, gangrenous necrosis^(^^34-36^^)^. Transvaginal ultrasound with Doppler flow study is the first-line imaging modality when ovarian torsion is suspected^(^^25,28^^)^. However, although the absence of flow on Doppler is highly suggestive of ovarian torsion, its presence does not exclude disease, because the ovaries have dual arterial supply^(^^34^^)^. Therefore, ovarian torsion can be a challenging diagnosis to make with ultrasound, particularly in subacute or intermittent cases; as such, MRI may be required for better evaluation^(^^25,35,36^^)^. Gadolinium-enhanced sequences are helpful, and the absence of parenchymal enhancement is a clue for the diagnosis^(^^32,34,35^^)^. In the affected ovary, hemorrhagic infarction, cytotoxic (cellular) edema, and blood clots from venous thrombosis cause restricted diffusion, with high SI at high b values and low ADCs^(^^21,25,34-36^^)^.

## TUBO-OVARIAN ABSCESS

**Figure 4:**
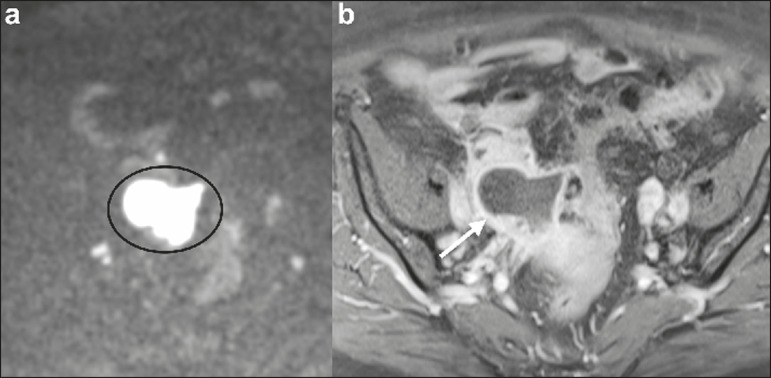
MRI of a female patient with tubo-ovarian abscess. **a**: Axial DWI at b = 1000 s/mm^2^ showing an area with high SI (circle). **b**: Axial T1W image with fat suppression after intravenous gadolinium-based contrast administration showing that this area did not enhance but exhibited a diffusely thickened wall that enhanced avidly (arrow).

Tubo-ovarian abscess is a condition within the wide spectrum of pelvic inflammatory disease^(^^26,32,38^^)^. Morphological MRI usually shows a complex cystic mass with ill-defined borders, thickened walls, and thickened septa, with low SI on T1W images and heterogeneously high SI on T2W images, which enhance after gadolinium-based contrast administration (Figure 4). As can be seen in Figure 4, the cystic component can present with low to slightly high SI on T1W images and slightly low to high SI on T2W images^(^^26,27,32,38^^)^. There are studies-such as those conducted by Li et al.^(^^27^^)^ and Oto et al.^(^^39^^)^-that advocate for the addition of DWI sequences to the morphological MRI protocols, because DWI improves their accuracy in diagnosing pelvic inflammatory disease and tubo-ovarian abscess, particularly if the use of a contrast agent is contraindicated^(^^25-27,38,39^^)^. The content of a tubo- ovarian abscess is pus-a viscous fluid that consists of bacteria, inflammatory cells, cellular debris, necrotic tissue (with coagulative necrosis), and proteinaceous plasma with high cellularity. Therefore, the higher the viscosity of the pus is, the higher will be the SI in DWI (Figure 4) and the lower will be the SI on the ADC map-although the restricted diffusion may be misleading, the lack of contrast enhancement indicates that it is pus and not a solid mass^(^^5,7,14,16,19,25-27,38-40^^)^. Consequently, when an area with restricted diffusion is depicted in the adnexa of a symptomatic patient with acute pelvic pain and fever, with simultaneous high to intermediate SI on T2W images and no enhancement after contrast administration, it is very likely an abscess^(^^12,27^^)^. Nevertheless, false negatives can occur in cases of chronic abscess, abscesses smaller than 1 cm in diameter, and abscesses under antibiotic therapy^(^^40^^)^.

## BLOOD-FILLED CYSTS

**Figure 5:**
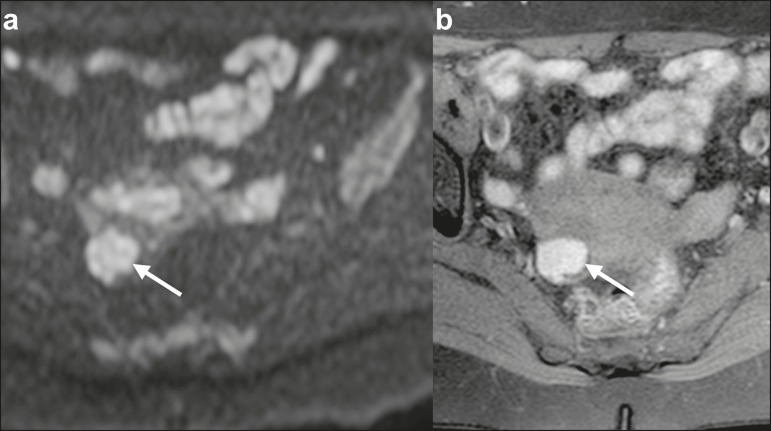
MRI of a female patient with endometrioma. **a**: Axial DWI at b = 1000 s/mm^2^ showing a right adnexal mass with relative high SI (arrow). **b**: Axial T1W images with fat suppression showing that the mass is spontaneously hyperintense (arrow).

**Figure 6:**
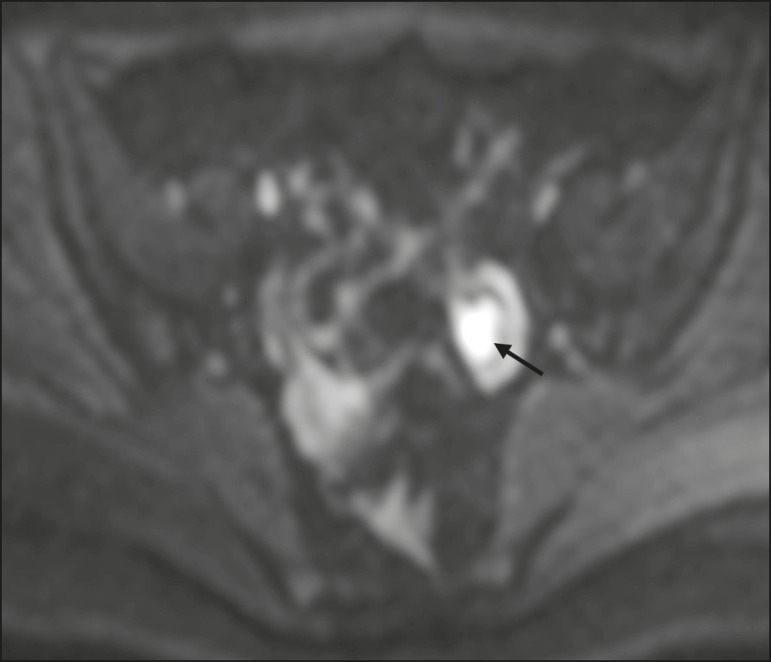
MRI of a female patient with a hemorrhagic cyst. Axial DWI at b = 1000 s/mm^2^ showing a left adnexal nodule with high SI (arrow).

Although transvaginal ultrasound can be useful in the diagnosis of ovarian endometrioma, MRI has been shown to have higher specificity^(^^15,31,37^^)^. On MRI, endometrioma typically appears as a cystic lesion with high SI on T1W images (Figure 5), with or without selective fat suppression, and relatively low SI on T2W images-the shading sign, which has been historically used to diagnose endometriomas^(^^31,32,41^^)^. However, that sign is not exclusive to endometriomas, and the "T2 dark spot" sign-consisting of T2 dark spots representing chronically retracted clots with high protein and hemoglobin content that exhibit T2 shortening-can therefore be useful in their diagnosis^(^^31,41^^)^. On DWI, endometriomas can exhibit low SI on ADC maps and high SI on DWI at b = 1000 s/mm^2^ (Figure 5), due to their thick proteinaceous and hemoglobin degradation products^(^^6-8,13,15,19,22,29,30,33,37,42^^)^. Because that can hamper the detection of malignant transformation, correlation with other sequences, either morphological or contrast-enhanced, is helpful whenever malignancy is suspected. Hemorrhagic cysts occur due to hemorrhage within a functional cyst and tend to reabsorb spontaneously. The sedimented blood of hemorrhagic cysts can show high SI on DWI at b = 1000 s/mm^2^ (Figure 6), with the corresponding low SI on ADC map, thereby mimicking malignant lesions^(^^5,9,30,37^^)^.

## MATURE CYSTIC TERATOMA

**Figure 7:**
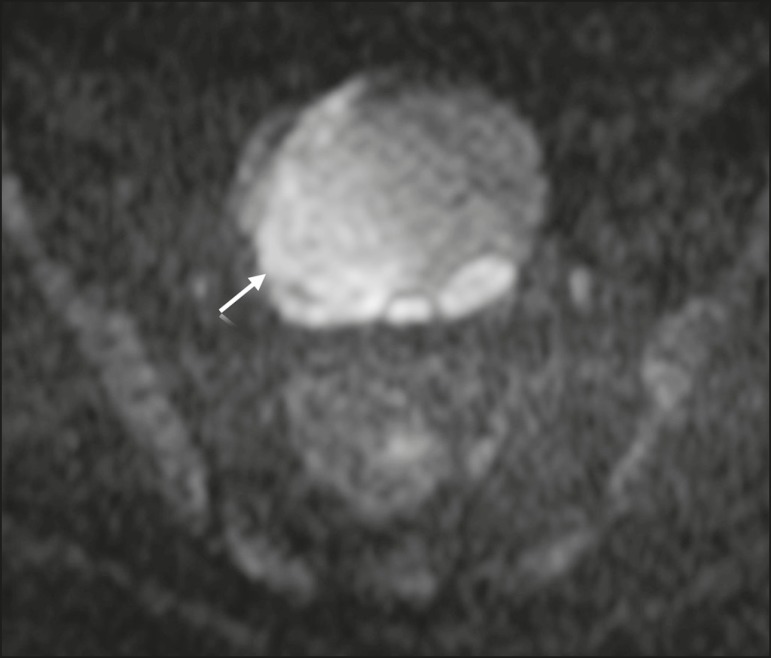
MRI of a female patient with mature cystic teratoma. Axial DWI at b = 1000 s/mm^2^ showing a tumor with areas of high SI (arrow).

Mature cystic teratomas are the most common ovarian tumor in women under 45 years of age and account for 95% of all ovarian germ-cell tumors^(^^32,43^^)^. They are composed of mature tissue from at least two of the three germ cell layers and are unilocular; in 88% of cases, they are filled with sebaceous material and are lined with keratinized squamous epithelium^(^^6,42-44^^)^. On MRI, there are typical features that allow the diagnosis of these tumors without biopsy. Their sebaceous content has high SI on T1W images, similar to that of retroperitoneal fat, becoming hypointense after selective fat suppression-that unique characteristic can be sufficient to establish its diagnosis^(^^13,19,32,42,43^^)^. On DWI, mature cystic teratomas containing keratinous materials have restricted diffusion-high SI on DWI at b = 1000 s/mm^2^ (Figure 7) and low SI on ADC maps^(^^6,7,13,19,21,22,26,29,33,37,42^^)^. Sala et al.^(^^19^^)^ and Motoshima et al.^(^^13^^)^ also stated that DWI can be helpful in diagnosing mature cystic teratomas with low fat content.

In rare cases, mature cystic teratoma can undergo malignant transformation and therefore show true restricted diffusion. The morphological correlation is mandatory, because malignant parietal nodules tend to show intermediate SI on T2W images and enhancement after gadolinium administration^(^^3,13,30,33^^)^.

## NON-EDEMATOUS FIBROMA

**Figure 8:**
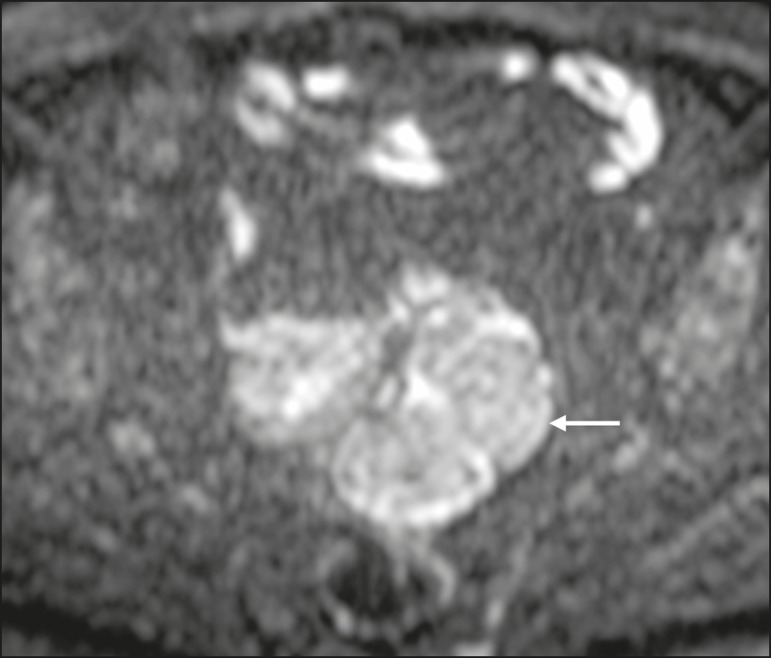
MRI of a female patient with fibroma. Axial DWI showing that the tumor has relatively high SI (arrow).

Fibromas are the most common ovarian sex cord-stromal tumors, occurring in premenopausal and postmenopausal women^(^^32,43^^)^. They are benign, have no theca cells, and do not exhibit estrogenic activity-being composed of whorled bundles of spindle-shaped fibroblasts and collagen^(^^43^^)^. They appear as solid masses and usually have a diameter of less than 10 cm. However, they can be quite large and can therefore resemble malignant neoplasms^(^^25,31^^)^. Non-edematous fibromas are composed of dense stromal proliferation and do not undergo edematous degeneration-which can occur in large fibromas^(^^22,45^^)^. Because non-edematous fibromas have high collagen content, they have low SI on T1W images and very low SI on T2W images^(^^32,43^^)^. When they reach large dimensions, fibromas can be misdiagnosed as pedunculated (subserosal) uterine or broad-ligament leiomyomas^(^^43^^)^. Because of their dense stromal proliferation, fibromas can show restricted diffusion, with high SI on DWI at b = 1000 s/mm^2^ (Figure 8) and low SI on ADC maps^(^^12,13,22,30,33^^)^. This pitfall can be avoided by assessing the very dark signal on T2W images.

## OTHER STRUCTURES WITH RESTRICTED DIFFUSION IN AND ADJACENT TO THE PELVIS

**Figure 9:**
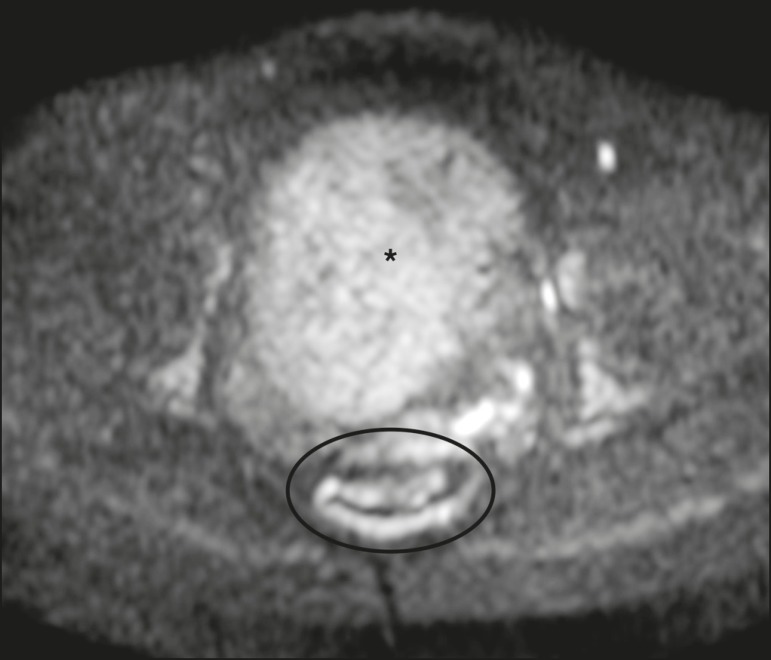
MRI, at the level of the rectum, of the same female patient depicted in Figure 2. Axial DWI showing a large hyperintense uterine tumor (asterisk)- the known cellular leiomyoma-and a posterior hyperintense ring (circle)-the rectal mucosa.

There are other pelvic structures that can show high SI on DWI at high b values with low SI on ADC maps and should not be mistaken for malignant tissue. In the urinary bladder, hematuria can be a potential pitfall, because some blood products have different SI on DWI and generally all have low ADCs-oxyhemoglobin (the predominant blood product at the hyperacute stage of hemorrhage) and extracellular methemoglobin (the predominant product at the late subacute stage) both show high SI on DWI at b = 1000 s/mm^2^(^46^**^-^**^48^**^)^**. That can lead to a false-positive diagnosis of malignancy, and the solution to overcome this potential pitfall is to compare DWI sequences with contrast-enhanced fat-suppressed T1W images, in which only solid tumor components will enhance. The normal rectal mucosa is hyperintense on DWI at any b value (Figure 9) and has low SI on ADC maps, because it has high cellular content and intact cell membranes. On axial images, it can appear as a complete or incomplete bright ring behind the uterus-this can be potentially confusing, and DWI findings must be correlated with those of the morphological MRI sequences^(^^12,14,49^^)^. The bone comprises two types of marrow^(^^50,51^^)^: red (rich in the hemoglobin of erythrocytes and their precursors); and yellow (rich in carotenoid derivates dissolved in adipocytes). Most pelvic MRI studies are performed in adults whose bone marrow has already partially converted to yellow marrow. In contrast, red marrow shows restricted diffusion because it is a highly cellular tissue. Diffuse marrow reconversion can occur in heavy smokers, long distance runners, obese women, and patients with hematological diseases, including anemia. The high SI on DWI sequences can lead to confusion between marrow reconversion and bone lesions^(^^7,46,50,51^^)^.

**Figure 10:**
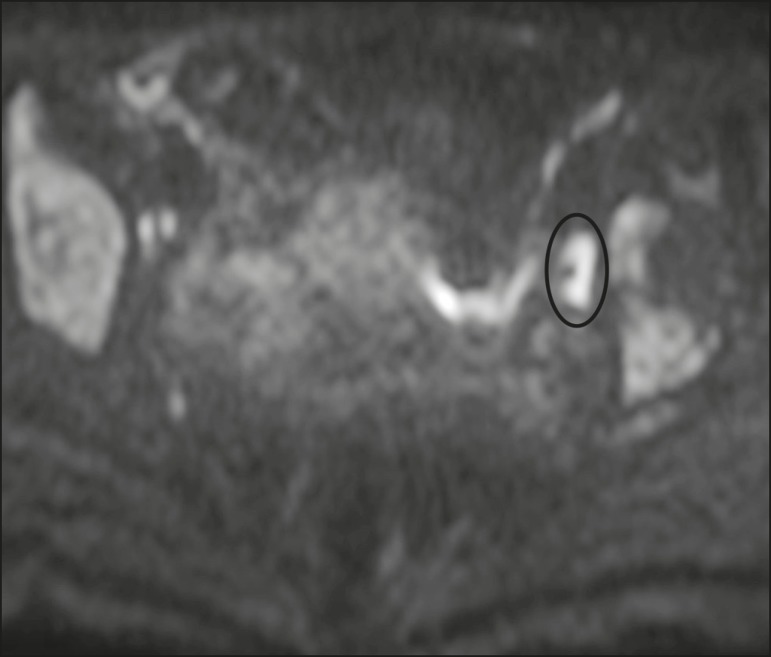
MRI of a pelvic lymph node in a female patient. Axial DWI showing a bright ovoid nodule in the topography of the obturator foramen (circle).

The assessment of lymph nodes is very important in the staging of pelvic tumors. The current criteria for lymph node metastases are based on the dimensions and morphology of the nodes^(^^4,13,14,19,52^^)^. The behavior of lymph nodes on DWI sequences is not completely understood, because they usually show high SI at all b values (Figure 10), whether or not they are affected by malignant tissue^(^^3,5,7,14,19,46,52^^)^. On ADC maps, some lymph nodes are bright-with the T2 shine-through effect-and should not be considered worrisome. However, those with low SI on the ADC map and simultaneous high SI on DWI at high b values-with restricted diffusion are suspicious. However, there is no ADC cutoff value to determine which of these nodes are malignant or benign; DWI alone therefore cannot be used in order to predict malignant involvement of lymph nodes, and their restricted diffusion can become a pitfall^(^^3,5,13,52^^)^.

## CONCLUSION

DWI contributes functional and structural information about biological tissues, without the use of ionizing radiation or intravenous contrast administration. This MRI modality is gaining ever increasing importance in multiparametric MRI and is now used routinely. Many non-malignant lesions have high SI on DWI at b = 1000 s/mm^2^ and low SI on ADC maps, resembling the behavior of malignant neoplasms. The interpretation and correlation of DWI sequences with conventional T1W and T2W images is mandatory, given that DWI is considered only a complementary sequence. In fact, although DWI is a noninvasive sequence and its cost-effectiveness has been proven, T1W images with fat suppression before and after the intravenous administration of gadolinium-based contrast media remains a cornerstone in the characterization of lesions on pelvic MRI-we not only get a more detailed characterization of the lesion but can also establish its boundaries and perfusion. Therefore, although DWI can be quite useful in the detection of lesions, there are several pitfalls in the imaging of the female pelvis, because many benign lesions show restricted diffusion. Radiologists should be aware of those pitfalls, recognizing how normal tissues and benign conditions behave on DWI sequences.
